# Exploring the risk factors and clustering patterns of periodontitis in patients with different subtypes of diabetes through machine learning and cluster analysis

**DOI:** 10.2340/aos.v83.42435

**Published:** 2024-12-03

**Authors:** Anna Zhao, Yuxiang Chen, Haoran Yang, Tingting Chen, Xianqi Rao, Ziliang Li

**Affiliations:** a Affiliated Stomatology Hospital of Kunming Medical University, Kunming, Yunnan, China; b Yunnan Provincial Key Laboratory of Stomatology, Kunming, Yunnan, China

**Keywords:** Periodontitis, diabetes mellitus, consistent consensus, cluster A, machine learning, predictive modelling

## Abstract

**Aim:**

To analyse the risk factors contributing to the prevalence of periodontitis among clusters of patients with diabetes and to examine the clustering patterns of clinical blood biochemical indicators.

**Materials and methods:**

Data regarding clinical blood biochemical indicators and periodontitis prevalence among 1804 patients with diabetes were sourced from the National Health and Nutrition Examination Survey (NHANES) database spanning 2009 to 2014. A clinical prediction model for periodontitis risk in patients with diabetes was constructed via the XGBoost machine learning method. Furthermore, the relationships between diabetes patient clusters and periodontitis prevalence were investigated through consistent consensus clustering analysis.

**Results:**

Seventeen clinical blood biochemical indicators emerged as superior predictors of periodontitis in patients with diabetes. Patients with diabetes were subsequently categorized into two subtypes: Cluster A presented a slightly lower periodontitis prevalence (74.80%), whereas Cluster B presented a higher prevalence risk (83.68%). Differences between the two groups were considered statistically significant at a *p* value of ≤0.05. There was marked variability in the associations of different cluster characteristics with periodontitis prevalence.

**Conclusions:**

Machine learning combined with consensus clustering analysis revealed a greater prevalence of periodontitis among patients with diabetes mellitus in Cluster B. This cluster was characterized by a smoking habit, a lower education level, a higher income-to-poverty ratio, and higher levels of albumin (ALB g/L) and alanine aminotransferase (ALT U/L).

## Introduction

Periodontitis, a chronic inflammatory disease caused by plaque microorganisms, is now the leading cause of tooth loss in adults and not only poses a serious socioeconomic burden but also interacts with a wide range of systemic diseases [1]. The relationship between periodontitis and diabetes has been widely reported [2, 3], with chronic periodontitis considered the sixth most common complication of diabetes [4–6]. A study by Dhir et al. revealed that the overall prevalence of periodontitis in patients with and without diabetes was 72% and 27%, respectively [7]. Diabetes increases people’s risk of periodontitis by 34%, and the periodontal status of diabetic individuals is worse [8–10]. Additionally, diabetes can promote the development of periodontitis [11]. The current diagnosis of periodontitis usually relies on the detection of clinical parameters (alveolar bone loss and clinical attachment levels) [12,14]. However, before these parameters can be used to detect the disease, severe damage to the periodontal tissue may have already occurred [15]. Therefore, finding a simple, rapid, and reliable way to diagnose and monitor the development of periodontitis in patients with diabetes at an early stage and to conduct early preventive and personalized interventions is highly clinically important.

Blood biochemical tests are not only routine medical check-ups but also provide a more intuitive and precise reference for diagnosing specific diseases. The analysis of some unique biomarkers present in saliva and blood can further help estimate the speed of disease occurrence and development and facilitate timely monitoring of the patient’s condition by healthcare professionals [16–18]. There is limited research on the relationship between blood biochemical markers and periodontitis in people with diabetes, and new evidence suggests that the prevention and management of oral health problems in diabetic individuals can minimize the harmful effects of hyperglycemia, with significant benefits for adults with diabetes [19–21]. Therefore, a total of 50 clinical and blood biochemical factors that may be associated with the prevalence of periodontitis in patients with diabetes were collected in this study; these factors included demographic characteristics, diseases, and lifestyles that may be associated with diabetes mellitus and periodontitis, as well as standard blood biochemical test indices [13, 22]. These factors are shown in Schedule 1. The machine learning algorithm was used to develop predictive models and perform consensus clustering analysis, which in turn were employed to identify populations of patients with diabetes at high risk for the occurrence of periodontitis and to make clinical decisions for patient management. This was done to provide targeted oral health education and develop a comprehensive periodontal treatment program. The aim of this study was to provide reference information for periodontal disease prevention in patients with diabetes, as well as for early clinical diagnosis and treatment.

**Figure 1:**
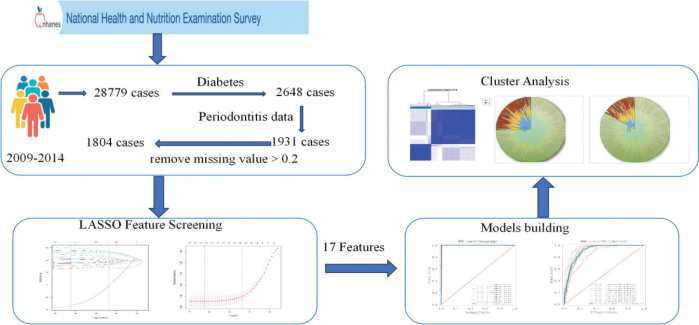
Flow chart of the study.

## Information and methods

### Data downloading and processing

**Figure 2:**
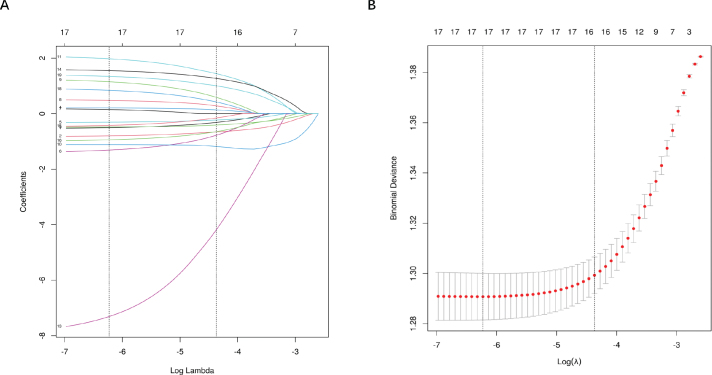
LASSO regression feature selection: coefficient profiles for Lasso regression (A) and plot of the optimal tuning parameter (λ) determined by 10-fold cross-validation of the Lasso regression (B).

The National Health and Nutrition Examination Survey (NHANES) is a multistage, nationally representative survey of the US noninstitutionalized population that has been conducted on a 2-year cycle since 1999, with data collected through interviews and physical examinations [23]. The Ethics Review Board of the CDC’s National Center for Health Statistics approved the protocol for collecting oral health data, and all survey participants provided written informed consent [24]. Since the NHANES collected periodontal examination data reflecting periodontal health (periodontal pockets, periodontal atrophy, and loss of attachment) from participants aged 30 years and older only from 2009 to 2014, data from three cycles, that is, 2009–2010, 2011–2012, and 2013–2014, with a total of 28,779 participants, were used in this study (Figure 2) . This study followed the Strengthening the Reporting of Observational Studies in Epidemiology (STROBE) guidelines [25]. The data analyzed were publicly available and therefore did not require any Institutional Review Board approval.

The study interviews included questions regarding demographic data (sex, age, race, marital status, pregnancy status, income-to-poverty ratio, frequency of alcohol consumption, sleep habits, job stress, dietary practices, etc.), education level, and medical history (diabetes, hypertension, hyperlipidemia, osteoporosis, etc.). The clinical examination included medical and dental evaluations and laboratory tests. The participants considered to have diabetes were individuals who self-reported having diabetes, had a glycated hemoglobin level of ≥6.5%, or were taking oral hypoglycemic medication or insulin [26]; there were a total of 2,648 participants, with 1,931 adults aged 30 years or older who underwent a periodontal health check-up (periodontal pockets, periodontal atrophy, and loss of adhesion). To ensure data quality, 1,804 patients with diabetes aged 30 years and older with complete periodontal examination data were ultimately enrolled in this study after the exclusion of participants with ≥0.2 missing interview content data and clinical examination data from the overall survey. The diagnosis of periodontitis was based on the classification of periodontitis developed for the American Academy of Periodontology in collaboration with the Centers for Disease Control and Prevention (CDC), which is applicable to NHANES data according to Eke et al. [27]. Mild periodontitis was defined as follows: 2 adjacent attachment losses (ALs) ≥ 3 mm, ≥ 2 adjacent probing depths (PDs) ≥ 4 mm (not on the same tooth), or 1 PD ≥ 5 mm [27]. Based on this assessment, 1,401 of the 1,804 participants included had periodontitis, and 403 did not have periodontitis. Missing values in the raw data were filled in via multiple interpolation with the R language ‘mice 3.12.0’ software package, and the processed data were background corrected and normalized by using MinMaxScaler (the samples were scaled to [0, 1]).

### Statistical analysis

Statistical analyses were performed via SPSS 25.0 software. Normality tests were conducted via the Kolmogorov-Smirnov test. Continuous variables are herein presented as medians (interquartile ranges [IQRs]) and were compared via the Mann-Whitney U test. Categorical variables are expressed as *n* (%) and were compared via the chi-square test or Fisher’s exact test. Group comparisons were made through the independent samples t test or one-way ANOVA. Count data are presented as [*n* (%)], and measurements are expressed as (x ± s). The relationships between clinical and blood biochemical factors affecting patients with diabetes and periodontitis were analyzed via Spearman’s correlation analysis. A significance level of *p* < 0.05 was used.

### Feature screening

Some studies have shown that when the model incorporates too many influencing factors, problems such as overfitting, degradation of the generalization ability, and an increase in covariance among independent variables may occur, making the model coefficients difficult to interpret [28]. In recent years, to avoid these problems and formulate simple and easy-to-explain clinical models, researchers have advocated the use of scientific statistical methods to screen variables before modeling when the model contains more variables [29, 30]. This is done to simplify the model as much as possible and improve its statistical efficiency and interpretability [31]. The traditional single-factor analysis screening results reflect only whether there are statistical discrepancies in the relationships between single influential factors and the results, ignoring the covariance among multiple influential factors and the effects of complex interactions on the results [32–34]. The least absolute shrinkage and selection operator (LASSO) compresses some of the regression coefficients by constructing a penalty function, forcing the sum of the absolute values of the coefficients to be smaller than a certain fixed value [35]. It also sets some of the regression coefficients to zero to obtain a more refined model. This technique can improve the prediction accuracy and explanatory power of the model. LASSO is currently one of the most widely used feature selection techniques [36, 37]. The basic principle is to introduce the L1 regularization term on the basis of the ordinary least squares method to achieve feature selection and coefficient sparseness in the model by minimizing the objective function. The LASSO regression objective function is as follows: minimize ||Y – X β||^2 + λ||β||₁, where Y is the observation value vector, X is the feature matrix, β is the regression coefficient vector to be estimated, and λ is a hyperparameter that controls the strength of regularization. The L1 regularization term λ||β||₁ plays a key role in the objective function. This term introduces sparsity and the coefficients of some features are reduced to zero, thereby achieving automatic feature selection. Therefore, LASSO regression can not only make predictions but also identify features that have an important impact on the target variable. The data were balanced via the SMOTE (Synthetic Minority Over Sampling Technique) method before feature selection so that the ratio of minority to majority categories was 1:1. The final matching result was periodontitis (1.0) = 1,401 patients with diabetes with periodontitis and periodontitis (0.0) = 1,401 patients with diabetes without periodontitis. LASSO regression was then used for feature selection to build this LASSO regression model on a binomial basis (R4.2.3 version: glmnet4.1.8 software package).

### ML modeling and development

Machine learning methods can be categorized into several types, including supervised learning, unsupervised learning, semi-supervised learning, and reinforcement learning. Supervised learning utilizes labeled training data to train models that are capable of making predictions on new data. In contrast, unsupervised learning automatically identifies patterns and regularities from unlabeled data. Semi-supervised learning combines supervised and unsupervised learning techniques, while reinforcement learning develops optimal behavioral strategies through trial and error. In this study, predictive models were developed and compared using nine commonly employed representative algorithms from the domain of supervised learning. The methods used to construct the predictive models include XGBoost, Logistic Regression, LightGBM, Random Forest, AdaBoost, Multi-Layer Perceptron, Support Vector Machines, k-Nearest Neighbours, and Naive Bayesian classification algorithm. All the prediction models were implemented via Python 3.7, with the packages ‘xgBoost 1.2.1’ for XGBoost, ‘lightgbm 3.2.1’ for XGBoost, and ‘skkbm’ for the remaining models. The package for XGBoost is ‘lightgbm 3.2.1’, and the package for the remaining models is ‘skLearning 0.22.1’. The bootstrap resampling technique was used to train and classify the ML models. The potential predictor variables selected in the feature selection process of LASSO regression were used in the predictive models. Patients were randomly divided into training and testing groups at an 8:2 ratio. The validation dataset was used to evaluate and compare the performance of each model. The ability of the model to predict periodontitis development in patients with diabetes was evaluated in terms of the area under the working characteristic curve (AUROC) of the subjects, accuracy, sensitivity, specificity, positive predictive value, negative predictive value, and F1 score. Model calibration was assessed via a ‘prediction versus observation’ plot for periodontitis incidence.

### Model optimization and evaluation

To ensure the stability of the model, tenfold cross-validation was used to evaluate its predictive ability. The training set was randomly divided into 10 groups, with eight groups used for training and two groups used for validation in each iteration. During training, 20% of the datasets from the training set were randomly selected to test the model’s performance. The models were then quantified individually via receiver operating characteristic (ROC) curve analysis, and their prediction accuracy was evaluated via area under the curve (AUC) and calibration plots. Clinical usefulness and net benefit were assessed via decision curve analysis (DCA). Shapley additive explanation (SHAP), a feature-importance metric based on game theory, was used to calculate the contribution of each feature to the model output. The SHAP was used to assess feature importance, with higher absolute SHAP values indicating features with the greatest impact on the model’s predictive scores. The distribution of feature values and their correlation with model predictions were also assessed, and the most reliable model was selected to construct a clinical prediction model for predicting the occurrence of periodontitis in patients with diabetes via blood test indices. An online web calculator was developed for applying the model and predicting the likelihood of periodontitis in specific individual patients with diabetes.

### Consensus clustering analysis

The K-means clustering algorithm is an unsupervised learning algorithm that is commonly used to divide the data set into K clusters. The K-means clustering algorithm can classify patients with similar disease risks into the same group, identify the characteristics of different disease risk patterns in patient groups, and help conduct risk assessments and customize personalized treatment plans. This study was based on the feature variables screened out by baseline analysis and LASSO regression, using the K-means clustering algorithm to calculate the distance between features and automatically aggregate and classify patients with diabetes to identify those with clinical and blood biochemical parameters at a high risk of periodontitis. The optimal number of groups (K) was determined by comparison with the flexed elbow method. The above statistical analyses were completed using the R package (version 4.2.3) and Python (version 3.11.4). The performance of clustering models is commonly evaluated by the silhouette coefficient and the Calinski-Harabasz index. The value of the silhouette coefficient ranges from –1 to 1. The closer the value is to 1, the better the model performance. The Calinski-Harabasz index ranges from 0 to +∞. The larger the value is, the better the model performance.

Continuous variables are expressed herein as the means ± standard deviations or medians ± IQRs. Differences between diabetes clusters were compared via the unpaired t test or Mann-Whitney U test. Categorical variables are expressed as absolute numbers (*n*) and relative frequencies (%) and were analyzed via the chi-square test or Fisher’s exact test. All the statistical analyses were performed via SPSS Statistics for Windows (version 25, IBM Corp., Armonk, NY, USA). Differences with *p* ≤ 0.05 were considered statistically significant.

## Results

### Statistical analysis

As shown in **Schedule** 1, 1,804 patients with diabetes were analyzed: 1,401 with periodontitis and 403 without periodontitis. The results indicate that age, race, education level, moderate leisure, high work intensity, income-to-poverty ratio, albumin (ALB g/L), alanine aminotransferase (ALT U/L), blood urea nitrogen (BUN mmol/L), total cholesterol (TC mmol/L), creatinine (Cr µmol/L), lactate dehydrogenase (LDH U/L), total protein (TP g/L), potassium (K mmol/L), osmolality (Osm mmol/kg), globulin (GLB g/L), and glycohemoglobin (GHb %) were among the 19 potential characteristics that were significantly different (*p* < 0.05) between the two groups of patients with diabetes with and without periodontitis. These characteristics may have an impact on the prevalence of periodontitis in patients with diabetes.

### Feature screening

After the data of 1804 patients (periodontitis (1.0) = 1401 patients in the periodontitis group of patients with diabetes and periodontitis (0.0) = 1401 patients in the nonperiodontitis group of patients with diabetes) were balanced via the SMOTE 1:1 (Synthetic Minority Oversampling Technique) method, the 19 potential characteristics (*p* < 0.05) were further screened via the LASSO regression algorithm. The results were further screened, as shown in Figure 2 (A and B), when the λ of the minimum mean square error in LASSO regression was 0.002. The 17 predictors (age, race, education level, moderate leisure, high work intensity, income-to-poverty ratio, drinking frequency, smoking status, ALB, ALT, TC, Cr, LDH, TP, K, Osm, and GHb levels) were considered the most valuable characteristics for developing a predictive model for periodontitis development in patients with diabetes, which could be used to construct predictive models.

### Predictive modeling

**Figure 3:**
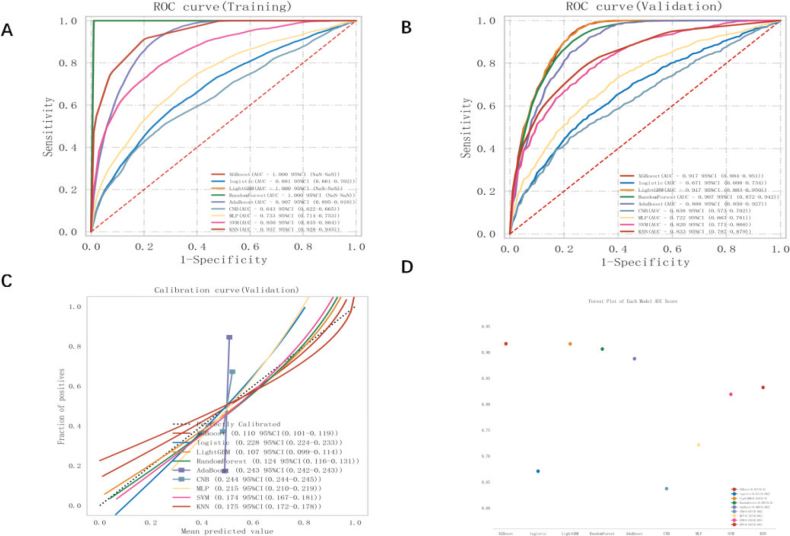
ROC curve analysis was conducted on nine machine learning models to predict the risk of periodontitis in diabetic patients. The analysis compared the performance of these models on the training set (A) and validation set (B). Calibrated typology plots (C) were used to evaluate the predictive models, and a forest plot (D) was created to display the AUC scores of the nine models.

The performance of the nine ML classification models for predicting the risk of periodontitis development in patients with diabetes was compared in the training set and validation set. This comparison is shown in Table 1 and Figure 3. Multiple evaluation metrics were employed to assess the performance of the prediction models. The ROC curves of the different ML models that predicted the presence or absence of periodontitis in patients with diabetes in the training and validation sets are shown in Figure 3 A and B. Calibration plots (Figure 3C) were used to determine the accuracy of the models. Furthermore, the ROC curve results for periodontitis prediction for each model are presented in the forest plot in Figure 3D. We observed that the XGBoost model performed better in both the training and validation sets, and it can be argued that XGBoost is the best model choice for this dataset and performs best in predicting the occurrence of periodontitis in patients with diabetes.

**Table 1:** Predictive performance of nine machine learning algorithms in the development of periodontitis in diabetic patients.

Models	AUC	Accuracy	Sensitivity	Specificity	PPV	NPV	F1 score	Kappa
XGBoost	0.917 (0.884–0.951)	0.803 (0.786–0.820)	0.719 (0.687–0.752)	0.887 (0.869–0.904)	0.864 (0.846–0.882)	0.761 (0.741–0.781)	0.784 (0.763–0.806)	0.606 (0.572–0.640)
logistic	0.671 (0.608–0.734)	0.628 (0.612–0.644)	0.592 (0.576–0.607)	0.665 (0.641–0.688)	0.639 (0.621–0.657)	0.619 (0.605–0.633)	0.614 (0.599–0.629)	0.256 (0.225–0.288)
LightGBM	0.917 (0.883–0.950)	0.834 (0.819–0.849)	0.828 (0.813–0.843)	0.84 (0.815–0.865)	0.839 (0.818–0.861)	0.83 (0.817–0.843)	0.833 (0.819–0.847)	0.668 (0.639–0.697)
Random Forest	0.907 (0.872–0.942)	0.827 (0.809–0.845)	0.804 (0.786–0.821)	0.85 (0.822–0.879)	0.844 (0.818–0.871)	0.813 (0.797–0.828)	0.823 (0.806–0.840)	0.654 (0.618–0.690)
AdaBoost	0.888 (0.850–0.927)	0.811 (0.794–0.828)	0.882 (0.855–0.908)	0.74 (0.712–0.769)	0.773 (0.755–0.792)	0.864 (0.839–0.889)	0.823 (0.807–0.840)	0.622 (0.587–0.656)
CNB	0.638 (0.573–0.702)	0.61 (0.597–0.622)	0.482 (0.450–0.514)	0.737 (0.701–0.773)	0.651 (0.624–0.679)	0.588 (0.578–0.598)	0.551 (0.532–0.571)	0.219 (0.195–0.244)
MLP	0.722 (0.663–0.781)	0.664 (0.647–0.682)	0.662 (0.621–0.702)	0.667 (0.625–0.708)	0.667 (0.646–0.689)	0.665 (0.645–0.685)	0.662 (0.640–0.684)	0.328 (0.293–0.364)
SVM	0.820 (0.771–0.868)	0.733 (0.719–0.748)	0.712 (0.673–0.751)	0.755 (0.723–0.787)	0.746 (0.725–0.767)	0.726 (0.703–0.749)	0.726 (0.707–0.746)	0.467 (0.437–0.496)
KNN	0.833 (0.787–0.879)	0.747 (0.740–0.755)	0.632 (0.615–0.650)	0.862 (0.838–0.886)	0.823 (0.802–0.845)	0.701 (0.695–0.708)	0.714 (0.706–0.722)	0.495 (0.480–0.510)

AUC: area under the curve; PPV: positive predictive value; NPV: negative predictive value; XGBoost: extreme gradient boosting; LR: logistic regression; LightGBM: light gradient boosting, RF: random forest; AdaBoost: AdaBoost Classifier; CNB: Complement NB; MLP: multi-layer perceptron; SVM: support vector machine; KNN: k-nearest neighbors.

**Figure 4:**
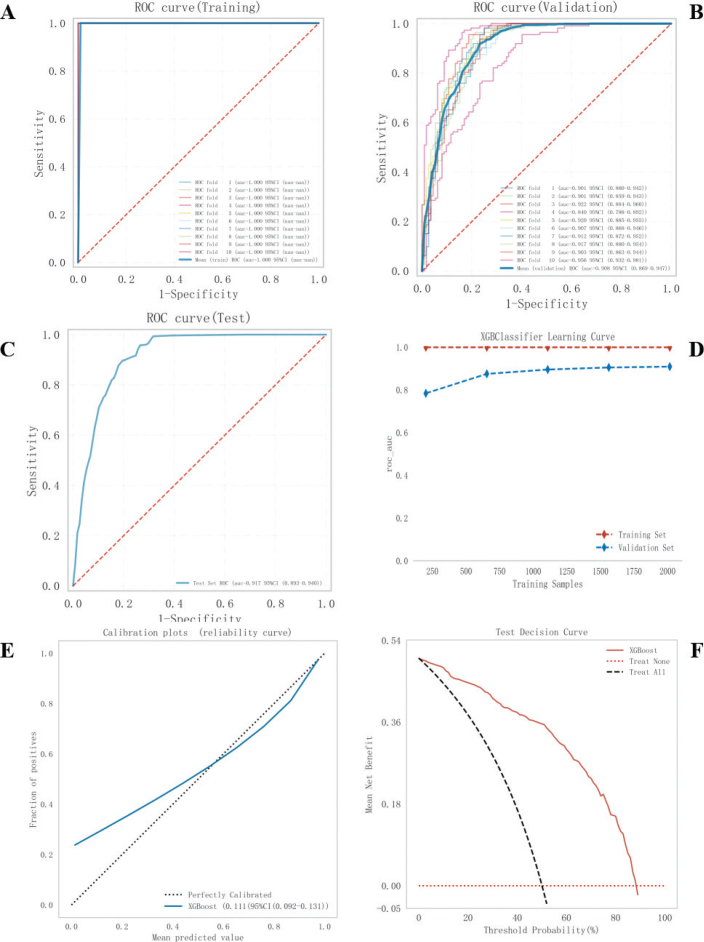

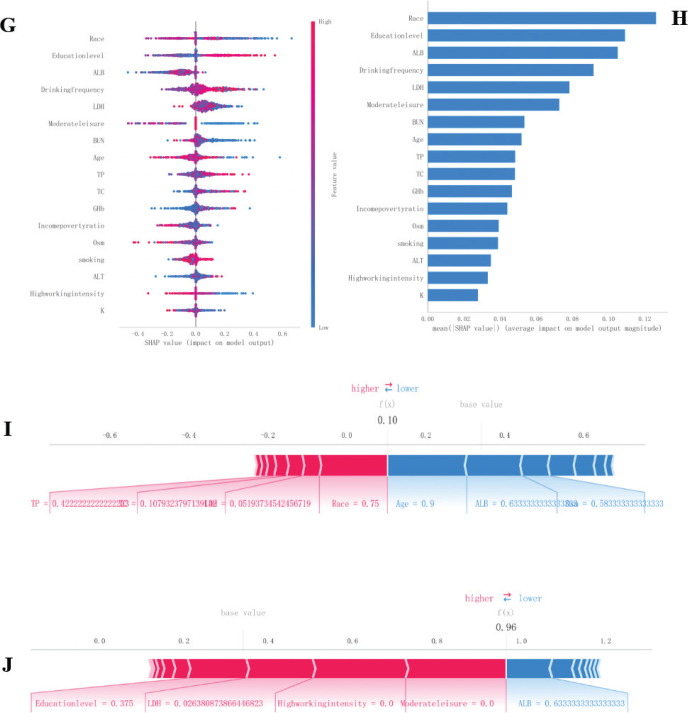
Construction and evaluation of the XGBoost model. (A-C) XGBoost ROC curves using 10-fold cross-validation on the training set (A), validation set (B), and test set (C). (D) Machine learning curves and (E) Calibration plots for XGBoost. (F) Decision curve analysis plot. (G–J) Feature importance SHAP summary charts and bar graphs. (G) The dot plots on the left indicate the direction of the contribution of each value of each variable, with red representing the larger value and blue representing the smaller value. (H) The bars on the right represent the importance of the variables and their overall contribution to the model predictions. (I), (J) SHAP score explains the predicted risk of periodontitis prevalence in two diabetic subjects.

**Figure 5:**
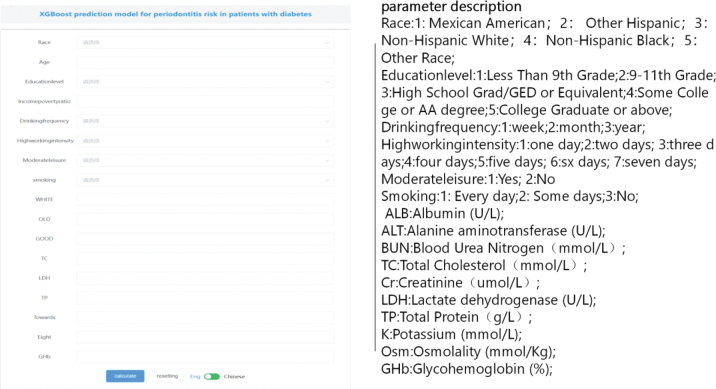
XGBoost constructs an online network calculator to predict the risk of periodontitis in diabetic patients.

The test set consisted of 560 cases (20.00%), which were randomly selected from a sample dataset of 2**,**802 cases (1**,**401 with periodontitis and 1**,**401 without periodontitis). The remaining samples were used for the training set in a 10-fold cross-validation. The XGBoost algorithm was used to predict the risk of patients with diabetes developing periodontitis. In the final test set, the model had an AUC of 0.9165 and an accuracy of 0.8021, demonstrating the best predictive performance, as shown in Table 2 and Figure 4A–C. The learning curve (Figure 4D) indicated that the model successfully predicted the risk of periodontitis development in patients with diabetes. The difference in error between the training and validation sets converged as the number of training samples increased, as shown in the learning curve. The calibration plot (Figure 4E) demonstrated that the XGBoost model accurately predicted the probability of the observed incidence of periodontitis in patients with diabetes. Additionally, a decision plot (Figure 4F) was constructed via a DCA, which showed the net gain in threshold probability on the basis of the model**’**s output decision. To further explain our model, we analyzed the results of the XGBoost algorithm via SHAP, as shown in Figure 4G. This analysis revealed the relationship between high and low eigenvalues, as well as the SHAP values in the dataset. Each point on the plot represents the eigenvalue and SHAP value for each patient. Figure 4H displays bar charts indicating the importance of the 17 features assessed via the SHAP values. The impact of each feature on the predictive model is represented by the bars with the mean absolute SHAP value. Figure 4I and J provide an individual-level breakdown of how these model features led to changes in predicted periodontitis risk scores for two example patients with diabetes. These figures identify which feature values strongly influenced the final risk prediction score by either increasing or decreasing it. On the basis of the feasibility of using the XGBoost algorithm and the 17 predictors mentioned above to predict the risk of periodontitis development in patients with diabetes, an online network calculation prediction model was developed, as shown in Figure 5.

**Table 2:** Performance of XGBoost model in predicting the risk of periodontitis in diabetic patients.

Model	AUC	Accuracy	Sensitivity	Specificity	PPV	NPV	F1 score
Training set	1.000 (NaN-NaN)	1.000 (1.000–1.000)	0.999 (0.999–0.999)	1.000 (1.000–1.000)	1.000 (1.000–1.000)	0.999 (0.999–0.999)	1.000 (1.000–1.000)
Validation set	0.908 (0.869–0.947)	0.784 (0.761–0.807)	0.683 (0.650–0.716)	0.884 (0.860–0.908)	0.855 (0.829–0.882)	0.737 (0.714–0.760)	0.908 (0.869–0.947)
Test set	0.917 (0.893–0.940)	0.802	0.705	0.9	0.876	0.752	0.781

### Consensus cluster analysis

**Figure 6:**
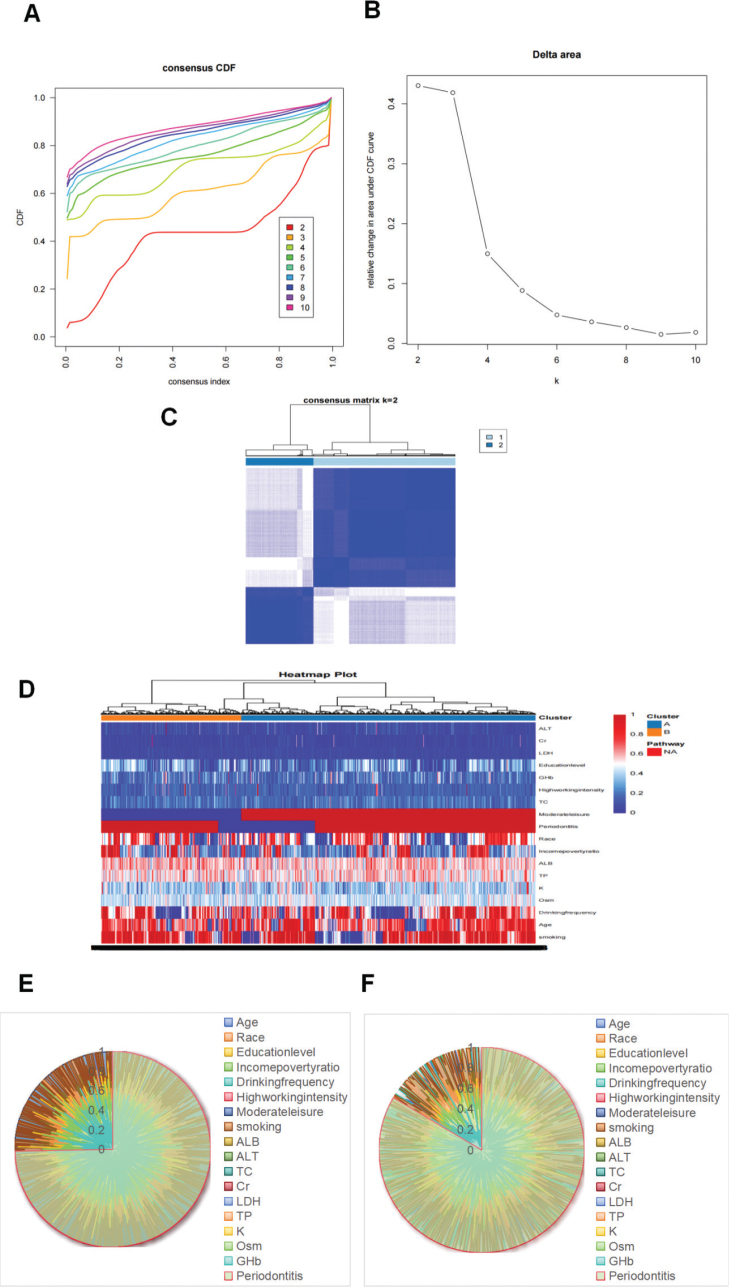
Consensus clustered cumulative distribution function (CDF) for (A) *k* = 2–10; relative change in the area under the CDF curve for (B) *k* = 2–10; heatmap of the cooccurrence matrix of the sample of diabetic patients for (C) *K* = 2. Heat map of cluster analysis results for (D). Diabetes subtype family 1 radar map for (E). Diabetes subtype family 2 radar map for (F).

To further analyze the prevalence of periodontitis in patients with diabetes, a consensus clustering algorithm was used in this study to identify 1,804 samples of patients with diabetes (with periodontitis: *N* = 1,401, without periodontitis: *N* = 403) with periodontitis. The optimal number of clusters was found when *k* = 2 (Figure 6A and B), and the 1,804 diabetic patient samples were divided into two clusters: Cluster A (*n* = 1,222) and Cluster B (*n* = 582) (Figure 6C). The periodontitis and smoking status, education level, income-to-poverty ratio, ALB level, and ALT level significantly differed across the various diabetes subclusters, as shown in Table 3 and Figure 6D–F.

**Table 3:** Cluster analysis table of diabetes subtypes based on periodontitis.

Variant	Subcategory	Overview (*n* = 1,804)	Cluster A (*n* = 1,222)	Cluster B (*n* = 582)	*p*
Periodontitis, *n* (%)	0	403 (22.339)	308 (25.205)	95 (16.323)	<0.001
	1	1401 (77.661)	914 (74.795)	487 (83.677)	
Race, *n* (%)	1	314 (17.406)	219 (17.921)	95 (16.323)	0.125
	2	178 (9.867)	112 (9.165)	66 (11.340)	
	3	627 (34.756)	438 (35.843)	189 (32.474)	
	4	473 (26.220)	322 (26.350)	151 (25.945)	
	5	212 (11.752)	131 (10.720)	81 (13.918)	
Education level, *n* (%)	1.0	350 (20.278)	249 (21.392)	101 (17.972)	<0.001
	2.0	293 (16.976)	218 (18.729)	75 (13.345)	
	3.0	381 (22.074)	267 (22.938)	114 (20.285)	
	4.0	434 (25.145)	278 (23.883)	156 (27.758)	
	5.0	263 (15.238)	148 (12.715)	115 (20.463)	
	9.0	5 (0.290)	4 (0.344)	1 (0.178)	
Drinking frequency, *n* (%)	1.0	295 (33.258)	189 (33.275)	106 (33.229)	0.611
	2.0	207 (23.337)	127 (22.359)	80 (25.078)	
	3.0	385 (43.405)	252 (44.366)	133 (41.693)	
High working intensity, *n* (%)	1.0	171 (13.266)	110 (12.500)	61 (14.914)	0.421
	2.0	1071 (83.088)	742 (84.318)	329 (80.440)	
	3.0	11 (0.853)	7 (0.795)	4 (0.978)	
	4.0	4 (0.310)	3 (0.341)	1 (0.244)	
	5.0	17 (1.319)	10 (1.136)	7 (1.711)	
	6.0	6 (0.465)	2 (0.227)	4 (0.978)	
	7.0	9 (0.698)	6 (0.682)	3 (0.733)	
Moderate leisure, *n* (%)	1	582 (32.262)	0 (0.000)	582 (100.000)	nan
	2	1222 (67.738)	1222 (100.000)	0 (0.000)	
Smoking, *n* (%)	1.0	253 (27.772)	207 (31.846)	46 (17.625)	<0.001
	2.0	39 (4.281)	20 (3.077)	19 (7.280)	
	3.0	619 (67.947)	423 (65.077)	196 (75.096)	
Age, median [IQR]	nan	59.000 [43.000,68.000]	60.000 [43.000,69.000]	57.000 [44.000,68.000]	0.373
Income poverty ratio median [IQR]	nan	1.670 [0.950,3.310]	1.590 [0.920,3.140]	1.880 [1.050,3.580]	0.002
ALB, median [IQR]	nan	41.000 [39.000,43.000]	41.000 [39.000,43.000]	42.000 [40.000,44.000]	<0.001
ALT, median [IQR]	nan	22.000 [17.000,30.000]	21.000 [16.000,30.000]	23.000 [18.000,31.000]	0.002
TC, median [IQR]	nan	4.577 [3.827,5.456]	4.603 [3.827,5.482]	4.500 [3.827,5.353]	0.228
Cr, median [IQR]	nan	79.560 [65.420,99.890]	79.560 [64.530,100.780]	77.790 [66.300,97.240]	0.172
LDH, median [IQR]	nan	129.000 [112.000,147.000]	129.000 [113.000,148.000]	127.000 [112.000,145.000]	0.096
TP, median [IQR]	nan	71.000 [68.000,75.000]	71.000 [68.000,75.000]	72.000 [68.000,75.000]	0.886
K, median [IQR]	nan	4.100 [3.800,4.300]	4.100 [3.800,4.300]	4.000 [3.800,4.300]	0.616
Osm, median [IQR]	nan	282.000 [278.000,285.000]	282.000 [278.000,285.000]	281.000 [278.000,285.000]	0.099
GHb, median [IQR]	nan	6.900 [6.400,8.000]	6.900 [6.400,8.000]	6.900 [6.400,8.000]	0.642

Periodontitis (1: yes; 0: no), Race (1: Mexican American; 2: Other Hispanic; 3: Non-Hispanic White; 4: Non-Hispanic Black; 5: Other Race), Education Level (1: College Degree or Higher; 2:College Degree; 3: High School Graduate/GED or Equivalent; 4: 9th-11th grade; 5: Less than 9th grade; 9: Don’t Know), Drinking frequency(1: Week; 2: Month; 3:Year), High working intensity: days of a week, Moderate leisure (1: yes; 2: no), Smoking (1: daily; 2: occasional; 3: no). ALB: Albumin (g/L); ALT: Alanine aminotransferase (U/L); TC: Total cholesterol (mmol/L); Cr: Creatinine (umol/L); LDH: Lactate dehydrogenase (U/L); TP: Total protein (g/L); K: Potassium (mmol/L); Osm: Osmolality (mmol/Kg); GHb: glycated hemoglobin (%).

## Discussion

In this study, we found that the prevalence of periodontitis among patients with diabetes was as high as 77.66%. We established a predictive model for periodontitis risk in patients with diabetes and conducted a consistent consensus Cluster A analysis. For the risk of periodontitis in patients with diabetes, age, race, education level, moderate leisure, high work intensity, income-to-poverty ratio, alcohol consumption frequency, smoking status, and the levels of ALB, ALT, TC, Cr LDH, TP, K, Osm, and glycohemoglobin (GHb%) were predicted by a comprehensive assessment of 17 risk predictors. The periodontitis risk was significantly greater in diabetes patients in Cluster B (83.68%) than in those in Cluster A (74.80%). There were also significant differences in clinical and blood biochemical indices among patients with diabetes in both clusters. Diabetes Subcluster B, which had a higher risk of periodontitis, was characterized mainly by a smoking habit, a lower education level, a higher income-to-poverty ratio, and higher ALB and ALT levels.

Smoking is recognized as an important risk factor for periodontitis. However, in a study by Han et al. [38], no synergistic effect of smoking and diabetes mellitus on periodontitis was observed. In other words, the combined effect of diabetes mellitus and smoking on periodontitis did not appear to be significant. However, in the present study, there were significantly more patients with diabetes in Subcluster B who were habitual smokers (75.10%) than there were in Subcluster A (65.08%). These findings suggest that smoking in patients with diabetes also leads to an increased risk of periodontitis, which is similar to the results of previous studies [39–41].

Education level and the income-to-poverty ratio can reflect an individual’s socioeconomic status to some extent. In this study, patients with diabetes with lower education levels were at greater risk of periodontitis, which is consistent with the findings of Walther and Zini et al. [42, 43]. Socioeconomic status inequality is associated with oral health inequality [39, 44, 45]. The study also revealed that a higher income-to-poverty ratio in diabetes patients in Cluster B was associated with a greater risk of periodontitis development, as shown by Buchwald et al. [46]. By examining the relationship between socioeconomic status factors, systemic inflammation, and the progression of periodontitis, previous studies have shown that a low level of education and lower economic income factors can impact the development of periodontitis. However, some scholars have argued that education level and economic factors do not seem to be significantly associated with the risk of periodontitis [47, 48]. Therefore, there is inconsistency in the results of previous studies regarding their influence on the risk of periodontitis. The results of this study may have been influenced by the complex disease course of diabetes, and further investigation is needed to determine the influence of education level and the income-to-poverty ratio on the risk of periodontitis in patients with diabetes.

Cui et al. [49] compared salivary proteins between healthy and periodontitis populations and reported that the concentrations of TP, ALB, and GLB were greater in the saliva of patients with periodontitis than in the saliva of healthy individuals. Banu et al. [50] reported that the concentrations of ALT and aspartate aminotransferase (AST U/L) in the serum and saliva were significantly greater in individuals with periodontitis than in healthy individuals. Widita et al. [51] analyzed the relationships between hepatic enzyme levels and periodontal clinical parameters and concluded that elevated ALT levels may be associated with changes in periodontal clinical parameters [52]. The present study also obtained similar results from the analysis of blood standard biochemistry and the periodontitis prevalence in patients with diabetes [16]. Cluster B patients with diabetes with a higher risk of periodontitis were found to have higher levels of ALB and ALT, suggesting that elevated levels of ALB and ALT remain important predictive risk factors for periodontitis development in patients with diabetes.

Machine learning models are becoming increasingly popular in precision medicine [53]. In this study, the use of machine learning to construct a clinical prediction model with k-means clustering helped to identify homogeneous groups and revealed stronger associations between potential risk factors for periodontitis development in patients with diabetes mellitus. These findings suggest that constructing a clinical prediction model and a clustering model using machine learning can aid in identifying high-risk groups of diabetes mellitus patients for periodontitis development and, it is hoped, improve the prevention, identification, treatment, and management of this common disease.

The present study had a cross-sectional study design, and issues such as temporally related causality and some recall bias could not be avoided despite our best efforts. Additionally, because only the 2009–2014 dataset from the NHANES included full-mouth periodontal clinical examination measurements and because that dataset primarily pertained to the US population, with a lack of similarly large samples of complete datasets since then, the generalizability of the present results for predicting the risk of periodontitis and the clustering of the current diabetic population is limited.

## Conclusion

The periodontitis risk prediction model for patients with diabetes, which was constructed on the basis of machine learning, shows excellent performance and can be used in the clinic for individualized prediction of the risk of periodontitis development in patients with diabetes. The consistent consensus Cluster A analysis method can effectively reveal the relationship between clusters of patients with diabetes and the risk of developing periodontitis, providing more support and guidance for personalized medical treatment and precision therapy of periodontal inflammatory disease in patients with diabetes. The accompanying online risk calculator serves as an easy-to-use tool for physicians to develop precise prevention and treatment strategies. In the future, our aim is to integrate more case data with imaging, molecular, and genetic data to further improve the performance of our risk assessment model and validate it in additional studies covering a wider population.

## Supplementary Material

Exploring the risk factors and clustering patterns of periodontitis in patients with different subtypes of diabetes through machine learning and cluster analysis

## Data Availability

The datasets supporting the conclusions of this study are available in the NHANES database (https://www.cdc.gov/nchs/nhanes).

## Declarations

Ethics approval and consent to participate: Not applicable.

## References

[1] Global burden of 369 diseases and injuries in 204 countries and territories, 1990–2019: a systematic analysis for the Global Burden of Disease Study 2019. Lancet. 2020;396(10258):1204–22. 33069326 10.1016/S0140-6736(20)30925-9 PMC7567026

[2] Kassebaum NJ, Smith AGC, Bernabé E, Fleming TD, Reynolds AE, Vos T, et al. Global, regional, 1 and national prevalence, incidence, and disability-adjusted life years for oral conditions for 195 countries, 1990–2015: a systematic analysis for the global burden of diseases, injuries, and risk factors. J Dent Res. 2017;96(4):380–7. 10.1177/0022034517693566 28792274 PMC5912207

[3] Genco RJ, Sanz M. Clinical and public health implications of periodontal and systemic diseases: an overview. Periodontology 2000. 2020;83(1):7–13. 10.1111/prd.12344 32385880

[4] Jain P, Hassan N, Khatoon K, Mirza MA, Naseef PP, Kuruniyan MS, et al. Periodontitis and systemic disorder – an overview of relation and novel treatment modalities. Pharmaceutics. 2021;13(8):null. 10.3390/pharmaceutics13081175 PMC8398110 34452136

[5] Stöhr J, Barbaresko J, Neuenschwander M, Schlesinger S. Bidirectional association between periodontal disease and diabetes mellitus: a systematic review and meta-analysis of cohort studies. Sci Rep. 2021;11(1):13686. 10.1038/s41598-021-93062-6 34211029 PMC8249442

[6] Nibali L, Gkranias N, Mainas G, Di Pino A. Periodontitis and implant complications in diabetes. Periodontol 2000. 2022;90(1):88–105. 10.1111/prd.12451 35913467 PMC9805043

[7] Dhir S, Wangnoo S, Kumar V. Impact of glycemic levels in type 2 diabetes on periodontitis. Indian J Endocrinol Metab. 2018;22(5):672–7. 10.4103/ijem.IJEM_566_17 30294579 PMC6166544

[8] Chee B, Park B, Bartold PM. Periodontitis and type II diabetes: a two-way relationship. Int J Evid Based Healthc. 2013;11(4):317–29. 10.1111/1744-1609.12038 24298927

[9] Wu CZ, Yuan YH, Liu HH, Li SS, Zhang BW, Chen W, et al. Epidemiologic relationship between periodontitis and type 2 diabetes mellitus. BMC Oral Health. 2020;20(1):204. 10.1186/s12903-020-01180-w 32652980 PMC7353775

[10] Tanwir F, Altamash M, Gustafsson A. Effect of diabetes on periodontal status of a population with poor oral health. Acta Odontol Scand. 2009;67(3):129–33. 10.1080/00016350802208406 19367474

[11] Zheng M, Wang C, Ali A, Shih YA, Xie Q, Guo C. Prevalence of periodontitis in people clinically diagnosed with diabetes mellitus: a meta-analysis of epidemiologic studies. Acta Diabetol. 2021;58(10):1307–27. 10.1007/s00592-021-01738-2 34028620

[12] Kebede TG, Pink C, Rathmann W, Kowall B, Völzke H, Petersmann A, et al. Does periodontitis affect diabetes incidence and haemoglobin A1c change? An 11-year follow-up study. Diabetes Metab. 2018;44(3):243–9. 10.1016/j.diabet.2017.11.003 29249612

[13] He S, Wei S, Wang J, Ji P. Chronic periodontitis and oral health-related quality of life in Chinese adults: a population-based, cross-sectional study. J Periodontol. 2018;89(3):275–84. 10.1002/JPER.16-0752 29543997

[14] Fuller J, Donos N, Suvan J, Tsakos G, Nibali L. Association of oral health-related quality of life measures with aggressive and chronic periodontitis. J Periodontal Res. 2020;55(4):574–80. 10.1111/jre.12745 32232983

[15] Ebersole JL, Nagarajan R, Akers D, Miller CS. Targeted salivary biomarkers for discrimination of periodontal health and disease(s). Front Cell Infect Microbiol. 2015;5:62. 10.3389/fcimb.2015.00062 26347856 PMC4541326

[16] Koppolu P, Sirisha S, Mishra A, Deshpande K, Lingam AS, Alotaibi DH, et al. Alkaline phosphatase and acid phosphatase levels in saliva and serum of patients with healthy periodontium, gingivitis, and periodontitis before and after scaling with root planing: a clinico-biochemical study. Saudi J Biol Sci. 2021;28(1):380–5. 10.1016/j.sjbs.2020.10.016 33424320 PMC7783641

[17] Botelho J, Lyra P, Proença L, Godinho C, Mendes JJ, Machado V. Relationship between blood and standard biochemistry levels with periodontitis in Parkinson’s Disease patients: data from the NHANES 2011–2012. J Pers Med. 2020;10(3):null. 10.3390/jpm10030069 PMC7565163 32722393

[18] Hyvärinen E, Savolainen M, Mikkonen JJW, Kullaa AM. Salivary metabolomics for diagnosis and monitoring diseases: Challenges and possibilities. Metabolites. 2021;11(9):null. 10.3390/metabo11090587 PMC8469343 34564402

[19] Esteves Lima RP, Atanazio ARS, Costa FO, Cunha FA, Abreu LG. Impact of non-surgical periodontal treatment on serum TNF-α levels in individuals with type 2 diabetes: a systematic review and meta-analysis. J Evid-Based Dent Pract. 2021;21(2):101546. 10.1016/j.jebdp.2021.101546 34391555

[20] Artese HP, Foz AM, Rabelo MS, Gomes GH, Orlandi M, Suvan J, et al. Periodontal therapy and systemic inflammation in type 2 diabetes mellitus: a meta-analysis. PLoS One. 2015;10(5):e0128344. 10.1371/journal.pone.0128344 26010492 PMC4444100

[21] Karikoski A, Ilanne-Parikka P, Murtomaa H. Oral self-care and periodontal health indicators among adults with diabetes in Finland. Acta Odontol Scand. 2001;59(6):390–5. 10.1080/000163501317153257 11831490

[22] Sischo L, Broder HL. Oral health-related quality of life: what, why, how, and future implications. J Dent Res. 2011;90(11):1264–70. 10.1177/0022034511399918 21422477 PMC3318061

[23] Hsueh L, Wu W, Hirsh AT, De Groot M, Mather KJ, Stewart JC. Undiagnosed diabetes among immigrant and racial/ethnic minority adults in the United States: National Health and Nutrition Examination Survey 2011–2018. Ann Epidemiol. 2020;51(null):14–9. 10.1016/j.annepidem.2020.07.009 32739530 PMC11129874

[24] Centers for Disease Control and Prevention, National Center for Health Statistics. National Health and Nutrition Examination Survey: NCHS Research Ethics Review Board (ERB) approval - NHANES 2009-2010, NHANES 2011-2012, NHANES 2013–2014 [Internet]. Available from: https://www.cdc.gov/nchs/nhanes/irba98.Htm [cited 24-08-2022]

[25] Observational studies: Getting clear about transparency. PLoS Med. 2014;11(8):e1001711. 10.1371/journal.pmed.1001711 25158064 PMC4144975

[26] Sacks DB, Arnold M, Bakris GL, Bruns DE, Horvath AR, Lernmark Å, et al. Guidelines and recommendations for laboratory analysis in the diagnosis and management of diabetes mellitus. Diabetes Care. 2023;46(10):e151–99. 10.2337/dci23-0036 37471273 PMC10516260

[27] Eke PI, Page RC, Wei L, Thornton-Evans G, Genco RJ. Update of the case definitions for population-based surveillance of periodontitis. J Periodontol. 2012;83(12):1449–54. 10.1902/jop.2012.110664 22420873 PMC6005373

[28] Slinker BK, Glantz SA. Multiple linear regression: accounting for multiple simultaneous determinants of a continuous dependent variable. Circulation. 2008;117(13):1732–7. 10.1161/CIRCULATIONAHA.106.654376 18378626

[29] Zhang HH. Discussion of sure independence screening for ultra-high dimensional feature space. J Roy Stat Soc B. 2008;70(5):903. 10.1111/j.1467-9868.2008.00674.x PMC2709408 19603084

[30] Li J, Dan J, Li C, Wu R. A model-free approach for detecting interactions in genetic association studies. Brief Bioinform. 2014;15(6):1057–68. 10.1093/bib/bbt082 24273216 PMC4296135

[31] Harrell FE, Lee KL, Mark DB. Multivariable prognostic models: issues in developing models, evaluating assumptions and adequacy, and measuring and reducing errors. Stat Med. 1996;15(4):361–87. 10.1002/(SICI)1097-0258(19960229)15:4<361::AID-SIM168>3.0.CO;2-4 8668867

[32] Dowd WW, Renshaw GM, Cech JJ, Kültz D. Compensatory proteome adjustments imply tissue-specific structural and metabolic reorganization following episodic hypoxia or anoxia in the epaulette shark (Hemiscyllium ocellatum). Physiol Genom. 2010;42(1):93–114. 10.1152/physiolgenomics.00176.2009 PMC2888556 20371547

[33] Montero-Lobato Z, Ramos-Merchante A, Fuentes JL, Sayago A, Fernández-Recamales Á, Martínez-Espinosa RM, et al. Optimization of growth and carotenoid production by haloferax mediterranei using response surface methodology. Mar Drugs. 2018;16(10):null. 10.3390/md16100372 PMC6213265 30304770

[34] Cai H, Pang X, Dong D, Ma Y, Huang Y, Fan X, et al. Molecular decision tree algorithms predict individual recurrence pattern for locally advanced nasopharyngeal carcinoma. J Cancer. 2019;10(15):3323–32. 10.7150/jca.29693 31293635 PMC6603411

[35] Tibshirani R. Regression shrinkage and selection via the Lasso. J Roy Stat Soc B (Methodol). 1996;58(1):267–88. 10.1111/j.2517-6161.1996.tb02080.x

[36] Chen Q, Hu H, He Q, Huang X, Shi H, Cao X, et al. Evaluating the risk of developing hyperuricemia in patients with type 2 diabetes mellitus using least absolute shrinkage and selection operator regression and machine learning algorithm. Digit Health. 2024;10(null):20552076241241381. 10.1177/20552076241241381 38550266 PMC10976486

[37] Pei X, Qi D, Liu J, Si H, Huang S, Zou S, et al. Screening marker genes of type 2 diabetes mellitus in mouse lacrimal gland by LASSO regression. Sci Rep. 2023;13(1):6862. 10.1038/s41598-023-34072-4 37100872 PMC10133337

[38] Han DH, Lim S, Kim JB. The association of smoking and diabetes with periodontitis in a Korean population. J Periodontol. 2012;83(11):1397–406. 10.1902/jop.2012.110686 22376209

[39] Mikami R, Mizutani K, Aoyama N, Matsuura T, Suda T, Takeda K, et al. Income-related inequalities in the association of smoking with periodontitis: a cross-sectional analysis in Tokyo Metropolitan Districts. Clin Oral Invest. 2023;27(2):519–28. 10.1007/s00784-022-04747-9 36241924

[40] Baumeister SE, Freuer D, Nolde M, Kocher T, Baurecht H, Khazaei Y, et al. Testing the association between tobacco smoking, alcohol consumption, and risk of periodontitis: a Mendelian randomization study. J Clin Periodontol. 2021;48(11):1414–20. 10.1111/jcpe.13544 34472130

[41] Alharthi SSY, Natto ZS, Midle JB, Gyurko R, O’Neill R, Steffensen B. Association between time since quitting smoking and periodontitis in former smokers in the National Health and Nutrition Examination Surveys (NHANES) 2009 to 2012. J Periodontol. 2019;90(1):16–25. 10.1002/JPER.18-0183 30102767

[42] Walther C, Spinler K, Borof K, Kofahl C, Heydecke G, Seedorf U, et al. Evidence from the Hamburg City Health Study – association between education and periodontitis. BMC Public Health. 2022;22(1):1662. 10.1186/s12889-022-14096-7 36056348 PMC9438138

[43] Zini A, Sgan-Cohen HD, Marcenes W. Socio-economic position, smoking, and plaque: a pathway to severe chronic periodontitis. J Clin Periodontol. 2011;38(3):229–35. 10.1111/j.1600-051X.2010.01689.x 21198768

[44] Borrell LN, Crawford ND. Social disparities in periodontitis among US adults: the effect of allostatic load. J Epidemiol Commun Health. 2011;65(2):144–9. 10.1136/jech.2009.098269 19996354

[45] Khajavi A, Radvar M, Moeintaghavi A. Socioeconomic determinants of periodontitis. Periodontol 2000. 2022;90(1):13–44. 10.1111/prd.12448 35950737

[46] Buchwald S, Kocher T, Biffar R, Harb A, Holtfreter B, Meisel P. Tooth loss and periodontitis by socio-economic status and inflammation in a longitudinal population-based study. J Clin Periodontol. 2013;40(3):203–11. 10.1111/jcpe.12056 23379538

[47] Hakeem FF, Sabbah W. Is there socioeconomic inequality in periodontal disease among adults with optimal behaviours. Acta Odontol Scand. 2019;77(5):400–7. 10.1080/00016357.2019.1582795 30919709

[48] Bitencourt FV, Nascimento GG, Costa SA, Andersen A, Sandbæk A, Leite FRM. Co-occurrence of periodontitis and diabetes-related complications. J Dent Res. 2023;102(10):1088–97. 10.1177/00220345231179897 37448314

[49] Cui B, Yu Y, Yuan W, Zhou W, Zhou X, Zhang P. [Variations in protein concentration and albumin/globulin ratio of whole unstimulated saliva obtained from healthy people and patients with chronic periodontitis]. Hua Xi Kou Qiang Yi Xue Za Zhi. 2015;33(4):339–42. 26552233 10.7518/hxkq.2015.04.003 PMC7030449

[50] Banu S, Jabir NR, Mohan R, Manjunath NC, Kamal MA, Kumar KR, et al. Correlation of toll-like receptor 4, interleukin-18, transaminases, and uric acid in patients with chronic periodontitis and healthy adults. J Periodontol. 2015;86(3):431–9. 10.1902/jop.2014.140414 25345339

[51] Widita E, Yoshihara A, Hanindriyo L, Miyazaki H. Relationship between clinical periodontal parameters and changes in liver enzymes levels over an 8-year period in an elderly Japanese population. J Clin Periodontol. 2018;45(3):311–21. 10.1111/jcpe.12861 29266357

[52] Helenius-Hietala J, Suominen AL, Ruokonen H, Knuuttila M, Puukka P, Jula A, et al. Periodontitis is associated with incident chronic liver disease – a population-based cohort study. Liver Int. 2019;39(3):583–91. 10.1111/liv.13985 30300961

[53] Ghassib IH, Batarseh FA, Wang HL, Borgnakke WS. Clustering by periodontitis-associated factors: a novel application to NHANES data. J Periodontol. 2021;92(8):1136–50. 10.1002/JPER.20-0489 33315260

