# Supplementary material for: Exploring the risk factors and clustering patterns of periodontitis in patients with different subtypes of diabetes through machine learning and cluster analysis
Source: Acta Odontol Scand. 2024 Dec 3;83:42435. doi: 10.2340/aos.v83.42435 (PMC11633034; doi:10.2340/aos.v83.42435)
Supplement: Supplementary file 1 — Exploring the risk factors and clustering patterns of periodontitis in patients with different subtypes of diabetes through machine learning and cluster analysis [file AOS-83-42435-s1.pdf]

Supplementary material has been published as submitted. It has not been copyedited or typeset by Acta Odontologica Scandinavica.

Schedule 1

Statistical analysis of risk factors for periodontitis development in diabetic patients.

variant	subcategory	overview (n=1804)	Periodontitis 0 (n=403)	Periodontitis 1 (n=1401)	p
Sex ,n(%)	1	907(50.277)	192(47.643)	715(51.035)	0.230
	2	897(49.723)	211(52.357)	686(48.965)	
Educationlevel ,median[IQR]	nan	3.000[2.000,4.000]	3.000[2.000,4.000]	3.000[2.000,4.000]	<0.001
Age ,median[IQR]	nan	59.000[43.000,68.000]	64.000[46.000,74.000]	57.000[43.000,67.000]	<0.001
Race ,n(%)	1	314(17.406)	47(11.663)	267(19.058)	<0.001
	2	178(9.867)	31(7.692)	147(10.493)	
	3	627(34.756)	177(43.921)	450(32.120)	
	4	473(26.220)	108(26.799)	365(26.053)	
	5	212(11.752)	40(9.926)	172(12.277)	
Hypertension ,n(%)	1	1183(65.576)	285(70.720)	898(64.097)	nan
	2	619(34.313)	118(29.280)	501(35.760)	
	9	2(0.111)	0(0.000)	2(0.143)	
Historyofantihypertensivedrugs ,n(%)	1.0	1473(81.652)	347(86.104)	1126(80.371)	nan
	1.1	156(8.647)	26(6.452)	130(9.279)	
	1.2	63(3.492)	12(2.978)	51(3.640)	
	1.3	25(1.386)	3(0.744)	22(1.570)	
	1.4	15(0.831)	4(0.993)	11(0.785)	
	1.5	12(0.665)	1(0.248)	11(0.785)	
	1.6	4(0.222)	0(0.000)	4(0.286)	
	1.8	1(0.055)	0(0.000)	1(0.071)	
	1.9	1(0.055)	0(0.000)	1(0.071)	
	2.0	54(2.993)	10(2.481)	44(3.141)	
Familyhistoryofdiabetes ,n(%)	1	1138(63.082)	247(61.290)	891(63.597)	0.541
	2	611(33.869)	141(34.988)	470(33.547)	
	9	55(3.049)	15(3.722)	40(2.855)	
Moderateleisure ,n(%)	1	582(32.262)	95(23.573)	487(34.761)	<0.001
	2	1222(67.738)	308(76.427)	914(65.239)	

Sleepdisorder ,n(%)	1	614(34.035)	151(37.469)	463(33.048)	0.099
	2	1190(65.965)	252(62.531)	938(66.952)	
Healthyeatingindex ,n(%)	1	124(6.874)	28(6.948)	96(6.852)	nan
	2	310(17.184)	85(21.092)	225(16.060)	
	3	784(43.459)	165(40.943)	619(44.183)	
	4	470(26.053)	101(25.062)	369(26.338)	
	5	114(6.319)	24(5.955)	90(6.424)	
	9	2(0.111)	0(0.000)	2(0.143)	
Maritalstatus ,median[IQR]	nan	2.000[1.000,3.000]	2.000[1.000,3.000]	2.000[1.000,3.000]	0.345
PregnTancystatus ,median[IQR]	nan	2.000[2.000,2.100]	2.000[2.000,2.100]	2.000[2.000,2.100]	0.864
Incomepovertyratio ,median[IQR]	nan	1.718[1.000,3.180]	1.509[0.920,3.090]	1.810[1.020,3.220]	0.037
Drinkingfrequency ,median[IQR]	nan	2.200[1.800,2.700]	2.300[2.000,2.700]	2.200[1.700,2.800]	0.018
Highserumcholesterol ,median[IQR]	nan	1.400[1.000,2.000]	1.400[1.000,2.000]	1.300[1.000,2.000]	0.749
Highworkingintensity ,median[IQR]	nan	2.000[2.000,2.000]	2.000[2.000,2.000]	2.000[1.900,2.000]	0.001
Hourofsleep ,median[IQR]	nan	7.000[6.000,8.000]	7.000[6.000,8.000]	7.000[6.000,8.000]	0.360
smoking ,median[IQR]	nan	2.700[2.000,3.000]	2.900[2.000,3.000]	2.600[2.000,3.000]	0.046
smokingfrenquency ,median[IQR]	nan	10.700[8.600,13.900]	10.300[8.400,13.900]	10.800[8.600,13.900]	0.512
BMI ,median[IQR]	nan	31.220[27.300,36.300]	30.980[27.000,36.200]	31.280[27.380,36.380]	0.435
WaistCircumference ,median[IQR]	nan	107.700[98.200,118.800]	108.340[98.900,118.920]	107.500[98.000,118.800]	0.500
Osteoporosis ,median[IQR]	nan	2.000[1.900,2.000]	2.000[1.900,2.000]	2.000[1.900,2.000]	0.892
ALB ,median[IQR]	nan	41.000[39.000,43.000]	41.000[39.000,43.000]	41.000[39.000,44.000]	0.027
ALT ,median[IQR]	nan	22.000[17.000,30.000]	20.000[16.000,28.000]	22.000[17.000,31.000]	<0.001
AST ,median[IQR]	nan	23.000[19.000,28.000]	22.000[19.000,28.000]	23.000[19.000,28.000]	0.471
ALP ,median[IQR]	nan	69.000[56.000,86.000]	72.000[56.000,91.000]	69.000[56.000,85.000]	0.161
BUN ,median[IQR]	nan	5.000[3.930,6.430]	5.360[3.930,7.500]	4.640[3.930,6.070]	<0.001

Ca ,median[IQR]	nan	2.350[2.300,2.425]	2.350[2.300,2.425]	2.350[2.300,2.425]	0.498
TC ,median[IQR]	nan	4.577[3.827,5.456]	4.344[3.672,5.172]	4.655[3.879,5.534]	<0.001
HCO3 ,median[IQR]	nan	25.000[24.000,27.000]	25.000[23.000,27.000]	25.000[24.000,27.000]	0.187
Cr ,median[IQR]	nan	79.560[65.420,99.890]	83.100[68.070,108.730]	77.790[64.530,97.240]	<0.001
GT ,median[IQR]	nan	23.000[17.000,37.000]	22.000[16.000,36.000]	24.000[17.000,37.000]	0.312
GLU ,median[IQR]	nan	7.330[5.880,10.160]	7.160[5.830,9.830]	7.380[5.880,10.210]	0.264
Fe ,median[IQR]	nan	13.100[10.000,16.800]	12.700[9.500,16.300]	13.100[10.200,17.000]	0.153
LDH ,median[IQR]	nan	129.000[112.000,147.000]	133.000[116.000,150.000]	127.000[112.000,146.000]	<0.001
P ,median[IQR]	nan	1.195[1.066,1.324]	1.195[1.066,1.324]	1.195[1.098,1.324]	0.565
TBIL ,median[IQR]	nan	10.260[8.550,13.680]	10.260[8.550,13.680]	10.260[8.550,13.680]	0.196
TP ,median[IQR]	nan	71.000[68.000,75.000]	70.000[67.000,74.000]	72.000[68.000,75.000]	<0.001
TG ,median[IQR]	nan	1.716[1.163,2.642]	1.660[1.163,2.484]	1.716[1.163,2.698]	0.214
UA ,median[IQR]	nan	333.100[279.600,398.500]	345.000[279.600,416.400]	327.100[279.600,398.500]	0.062
NTa ,median[IQR]	nan	139.000[137.000,141.000]	139.000[138.000,141.000]	139.000[137.000,141.000]	0.064
K ,median[IQR]	nan	4.100[3.800,4.300]	4.100[3.900,4.400]	4.000[3.800,4.300]	0.005
Cl ,median[IQR]	nan	103.000[101.000,105.000]	103.000[102.000,105.000]	103.000[101.000,105.000]	0.096
Osm ,median[IQR]	nan	282.000[278.000,285.000]	282.000[279.000,286.000]	281.000[278.000,284.000]	0.002
GLB ,median[IQR]	nan	30.000[27.000,33.000]	29.000[26.000,33.000]	30.000[27.000,33.000]	0.013
GHb ,median[IQR]	nan	6.900[6.400,8.000]	6.700[6.300,7.700]	6.900[6.400,8.100]	0.005
FBG ,median[IQR]	nan	7.622[6.190,10.380]	7.380[6.110,9.990]	7.693[6.224,10.440]	0.162
PBG ,median[IQR]	nan	13.990[10.788,18.513]	13.888[10.550,17.926]	14.000[10.832,18.710]	0.310
INS ,median[IQR]	nan	102.606[73.224,146.064]	103.380[74.100,146.622]	102.300[73.104,145.746]	0.663

Data is displayed as mean ± standard deviation or median (interquartile range) or percentage.

Sex (1: Male; 2: Female), Race (1: Mexican American; 2: Other Hispanic; 3: Non-Hispanic White; 4: Non-Hispanic Black; 5: Other Race), Education Level (1: College Degree or Higher; 2:College Degree; 3: High School Graduate/GED or Equivalent; 4: 9th-11th grade; 5: Less than 9th grade; 9: Don't Know), Marital Status (1: Married; 2: Widowed; 3: Divorced; 4: Separated; 5: Unmarried; 6: Living with a partner; 77: Refused to answer), Pregnancy Status (1: Yes; 2: No; 3: Unable to Determine), Age, Ratio of Income to Poverty, Frequency of Drinking Alcohol (1: Weekly; 2: Monthly; 3: Yearly), High Blood Pressure (1: Yes; 2: No; 9: Don't know), History of prescription medication for hypertension (1: Yes; 2: No), High serum cholesterol levels (1: Yes; 2: No; 9: Don't know), Family history of diabetes (1: Yes; 2: No; 9: Don't know), Number of days of high-intensity work in a week, Moderate recreational and leisure exercise (1: Yes; 2: No), Number of hours of sleep, Sleep Difficulty (1: Yes; 2: No), Smoking (1: Daily; 2: Occasional; 3: No), Mean cigarettes smoked/day, Body Mass Index, Waist Circumference, Healthiness of Eating (1: Very good; 2: Good; 3: Good: 4: Normal: 5: Poor; 9: Don't know), Osteoporosis (1: Yes; 2: No; 9: Don't know), Albumin (g/L), Alanine aminotransferase (U/L), Aspartate aminotransferase (U/L), Alkaline phosphatase (U/L), Urea nitrogen (mmol/L), Calcium (mmol/L), Total cholesterol (mmol/L), Bicarbonate (mmol/L), Creatinine (umol/L), γ-Glucose (mmol/L), Iron (umol/L), Lactate dehydrogenase (U/L), Phosphorus (mmol/L), Total bilirubin (umol/L), Total protein (g/L), Triglycerides (mmol/L), Uric acid (umol/L), Sodium (mmol/L), Potassium (mmol/L), Total bilirubin (umol/L), Total protein (g/L), Triglycerides (mmol/L), Uric acid (umol/L), Sodium (mmol/L), Potassium (mmol/L), Glibenclamide (g/L), Glycated hemoglobin, Fasting blood glucose (mmol/L), 2-hour glucose (OGTT, mmol/L), Insulin (pmol/L), Periodontitis (1: Yes; 0: No); p-value < 0.5 : No); a P value of <0.05 was considered a statistically significant difference between the two groups.
